# The Impact of Cataract Surgery on Vision-Related Quality of Life and Psychological Distress in Monocular Patients

**DOI:** 10.1155/2021/4694577

**Published:** 2021-12-21

**Authors:** Xuepei Li, Jianqiang Lin, Zidong Chen, Guangming Jin, Danying Zheng

**Affiliations:** State Key Laboratory of Ophthalmology, Zhongshan Ophthalmic Center, Sun Yat-sen University, Guangzhou 510060, China

## Abstract

**Purpose:**

To determine the changes in vision-related quality of life and psychological distress after cataract surgery in monocular patients and to compared these with a control group of age- and gender-matched binocular patients.

**Methods:**

We enrolled 40 monocular patients and 40 binocular patients who underwent cataract surgery from August 2017 to December 2018. All participants undertook eye examinations and answered questionnaires (the National Eye Institute Visual Function Questionnaire, Self-rating Anxiety Scale, and Self-rating Depression Scale) before and after cataract surgery.

**Result:**

The monocular patients had significantly worse mean CDVA than the binocular patients before and after surgery. However, there was no significant difference between the increases gained by the two groups. Mean composite VFQ-25 scores of the monocular group were significantly lower than those of the binocular group before and after surgery, but the improvement experienced by the monocular group was statistically larger than the binocular group (37.20 ± 12.84 vs. 19.11 ± 5.13, *P* < 0.001). Mean standard SAS scores of monocular patients were significantly higher than those of binocular controls before and after surgery, while monocular patients experienced a significant greater decline of SAS scores (−9.41 ± 5.39 VS −3.84 ± 1.61, *P* < 0.001). Mean standard SDS scores of the monocular group were significantly higher than those of the control group before and after surgery, but the monocular group experienced a significantly greater decline of SDS scores following cataract surgery (−11.91 ± 6.38 VS −4.78 ± 1.79, *P* < 0.001). There was a significant correlation between the preoperative logMAR CDVA and both the postoperative logMAR CDVA (*r* = 0.63, *P* < 0.001) and the changes in the logMAR CDVA (*r* = −0.881, *P* < 0.001) for monocular patients. Linear regression analyses suggested that higher postoperative VFQ-25 scores had significant associations with better preoperative CDVA and the absence of systemic comorbidity (both *P* < 0.05). Age and ocular comorbidity were significantly associated with postoperative SAS scores (both *P* < 0.05). Age and systemic comorbidity remained significant impact factors for SDS scores (both *P* < 0.05).

**Conclusion:**

Monocular patients reported greater improvement in vision-related quality of life and greater decline in the level of anxiety and depression than binocular control subjects, despite having similar CDVA gains after cataract surgery. We argue that it is not a better choice for monocular patients to delay cataract surgery until the cataract is very advanced. A clear understanding of the impact of cataract surgery on vision-related quality of life and psychological distress in monocular patients is needed by ophthalmologists when making surgery decision.

## 1. Introduction

Monocular patients are not common in ophthalmic practice. Previous studies have reported a 3.27%–7.9% prevalence rate of unilateral blindness in China [1–3]. When a 1-eyed patient develops a cataract in the remaining eye, ophthalmologists are reluctant to recommend cataract surgery, often delaying cataract surgery as long as possible, because of concerns that the consequences of poor outcome from cataract surgery would be more serious for monocular patients than for patients with binocular vision.

Although the risks of surgery must always be considered, vision loss from cataract affects the quality of life of monocular patients more greatly than that of binocular patients [4], and patients may experience anxiety and depression while awaiting cataract surgery [5, 6]. All the adverse effects from cataract surgery must be weighed in the balance.

Many studies found that cataract surgery benefits patients through meaningful gains in visual function, quality of life, and reduction of psychological distress [7–10], nevertheless little is known regarding the impact of cataract surgery on vision-related quality of life and psychological distress in monocular patients. Although previous studies have reported outcomes of cataract surgery in monocular patients, such as corrected distance visual acuity, postoperative procedures, surgical complications, and visual function [4, 11, 12], and there have been no outcomes-based studies from which ophthalmologists can draw guidance to determine when to perform cataract surgery for monocular patients.

We undertook this prospective study to determine the visual outcomes and the changes in the quality of life and psychological distress after cataract surgery in monocular patients. In addition, we analyzed predictors of postoperative corrected distance visual acuity, postoperative quality of life, and postoperative psychological disturbances. Based on this knowledge, ophthalmologists can draw guidance to make appropriate surgery decisions and to direct psychological interventions to enhance patients' cooperation with surgery or follow-up, as well as to promote postoperative rehabilitation.

## 2. Methods

### 2.1. Study Design and Participants

This prospective comparative study enrolled participants who underwent cataract surgery from August 2017 to December 2018 in Zhongshan Ophthalmic Center, Sun Yat-sen University. We identified 40 patients who were monocular at the time of surgery. Individuals with monocular vision were diagnosed with irreversible blindness in the nonsurgical eye on the basis of corrected distance visual acuity (CDVA) worse than 1.3 LogMAR (20/400) or visual field loss to less than 10 degrees, based on WHO definitions. A cohort of binocular control patients, matched with monocular patients by age and gender, were also identified. Reasons for exclusion included psychiatric disorder or cognitive impairment that might affect cooperation or the psychological assessment, severe systemic disease, history of drug abuse, or previous cataract surgery. The study followed the tenets of the Declaration of Helsinki. All patient records and information from the medical database were anonymized and deidentified before being analyzed, and our study was approved by the institutional review board of Zhongshan Ophthalmic Center in Sun Yat-sen University (IRB-ZOC-SYSU).

### 2.2. Ophthalmic Examination and Procedure

Preoperatively, each patient was given a standard examination using ophthalmoscopy and slit-lamp biomicroscopy. Data recorded included demographic information, health status, reason for poor vision in the blind fellow, ocular comorbidities in the operated eye, and intraoperative and postoperative complications or procedures. Corrected distance visual acuity (CDVA) was scored as the total number of letters read correctly and was converted to logMAR (log_10_ minimum angle resolvable) corrected distance visual acuity.

All cataract surgeries were performed using phacoemulsification technique with posterior chamber intraocular lens (IOL) implantation. All the procedures were performed with topical anesthesia. Three experienced surgeons completed all surgeries.

After surgery, patients received routine postoperative care that typically consists of clinic visits and postoperative visual acuity (uncorrected and corrected) measurements at 1 day, 1 week, and approximately 30 days after surgery. Patients were instructed to complete the questionnaire and scales about 1 week preoperatively and 30 days postoperatively. Participants who could not complete the survey independently were provided with support from one trained investigator.

### 2.3. Assessment of Quality of Life

Assessments of vision-related quality of life were performed using the National Eye Institute Visual Function Questionnaire (NEI-VFQ-25), whose validity and reliability has been proven for use with cataract patients [7, 13]. The Chinese version of NEI-VFQ-25 was validated for Chinese patients [14]. The questionnaire consists of 25 questions, grouped into 12 subdomains, including one or more questions each. The subdomains include general health, general vision, ocular pain, near activities, far activities, social functioning, mental health, role difficulties, dependence, driving difficulties, color vision, and peripheral vision. The survey scores individual sections and the overall survey by weighed averages from 0 to 100. Complete visual disability would be a score of zero, and maximum visual function would be 100. The subscale scores were the average of one or more questions. The composite score of the NEI-VFQ-25 was the average of all the questions, except for the question about general health [13].

### 2.4. Assessment of Psychological Status

Individuals completed two standardized assessments measuring anxiety and depression symptoms. The Self-rating Anxiety Scale (SAS) is a 20-item, four-point Likert scale self-report assessment for anxiety symptoms [15]. The Self-rating Depression Scale (SDS) is a 20-item, four-point Likert scale self-report assessment for depression [16]. Item responses of both SAS and SDS are ranked from 1 to 4, with higher scores corresponding to more frequent symptoms. The raw total scores were converted to a 100-point scale by multiplying by 1.25. The Chinese versions of both scales have been validated and have been widely used to assess anxiety and depression associated with various diseases [17, 18].

### 2.5. Statistical Analysis

Statistical analysis was performed using SPSS software (SPSS for Windows, version 22.0, IBM-SPSS, Chicago, IL, USA). Variables were expressed as mean ±SD. We applied the chi-squared test or Fischer's exact test (for expected frequencies of less than 5) to test homogeneity with respect to proportions. Independent samples between the groups were compared using Student's *t*-test, and when the assumption of data was rejected, the Mann–Whitney test was applied. All *P* values were two-sided and were considered statistically significant if the values were less than 0.05.

Spearman's correlation coefficient was used to investigate the correlations between the pre- and postoperative logMAR CDVA and the relationship between the preoperative logMAR CDVA and the changes of logMAR CDVA in the operated eye. To examine the relationship between the various explanatory variables and the postoperative VFQ-25 composite scores and SAS and SDS scores, we performed multiple regression analyses of the data collected before surgery. The variables that were analyzed included age, gender, education level, residential environment, systemic comorbidity (yes/no), ocular comorbidity (yes/no), and preoperative logMAR CDVA in the operated eye.

## 3. Results

The study included 80 patients, with 40 being monocular and 40 being control subjects. The sociodemographic and other information are displayed in Table 1. The two groups were comparable with respect to age, gender, education level, and systemic comorbidities. However, the monocular group had significantly higher percentages of living in rural areas and greater number of eye comorbidities (both *P* < 0.05).

The preoperative, 1-month postoperative CDVAs for each group are shown in Table 2. Significant differences were found between groups in terms of the CDVA before and after surgery (all *P* < 0.05). Significant improvements in CDVA were observed at 1 month after surgery in both groups (both *P* < 0.05). However, no significant difference was found between the two groups with regard to CDVA changes after surgery (*P*=0.103).

**Figure 1:**
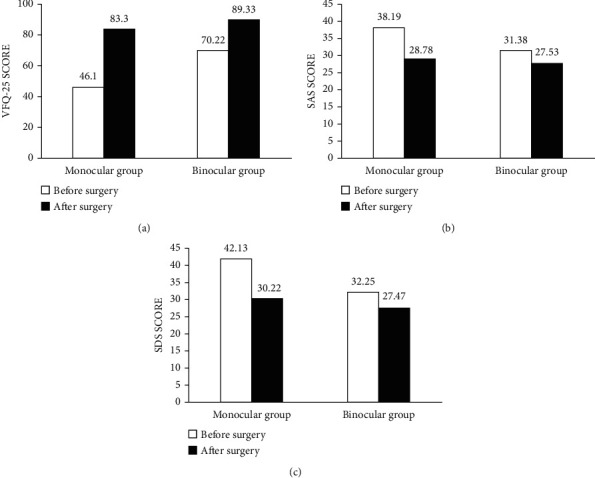
(a) Composite scores of the NEI-VFQ-25 for the monocular and binocular groups before and after surgery. (b) Comparison of the scores of self-rating anxiety scale between two groups before and after surgery. (c) Comparison of the scores of self-rating depression scale between two groups before and after surgery.

As shown in Figure 1(a), the mean composite score of the NEI-VFQ-25 for monocular patients before surgery was significantly lower than that of binocular patients (46.1 vs. 70.22, *P* < 0.001). The postoperative VFQ-25 composite score of the monocular group remained significantly lower than that of the control group (83.3 VS 89.33, *P* < 0.001). The monocular group gained a significantly greater improvement in the VFQ-25 composite score than did the binocular group after cataract surgery (*P* < 0.001). The mean VFQ-25 composite score for the monocular group improved 37.20 vs. 19.11 for the binocular group. Except for the driving subscale, monocular patients reported significantly greater improvements in all of subscales than did binocular patients (all *P* < 0.05).

With respect to comparisons between the two groups in terms of levels of anxiety and depression, the standard scores of the SAS and SDS for the two groups before and after surgery are presented in Figures 1(b) and 1(c). The monocular group had significantly higher SAS scores and higher SDS scores than the binocular group whether preoperative or postoperative (all *P* < 0.05). Moreover, monocular patients experienced significantly greater declines of SAS scores and SDS scores following cataract surgery than did the binocular patients (both *P* < 0.001). Based on the criteria of Zung, who stated that an SAS score ≥ 45 was the cutoff point for anxiety, only four monocular patients met this criterion before surgery. Considering the criteria of Zung, who stated an SDS score ≥ 50 was the cutoff point for anxiety, there were only five patients in the monocular group who met the criterion before surgery. Except for one monocular patient, all patients in both groups experienced declines of SAS scores after surgery. Except for one monocular patient and one control subject, all patients' SDS scores decreased after surgery.

**Figure 2:**
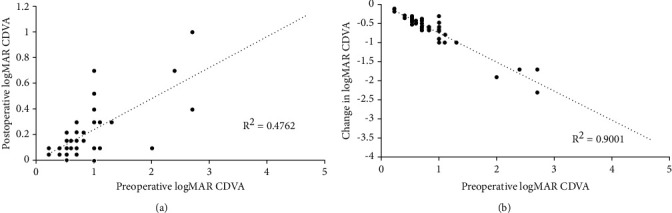
(a) Preoperative logMAR CDVA versus postoperative logMAR CDVA for the monocular group. (b) Preoperative logMAR CDVA versus changes in the logMAR CDVA for the monocular group.

As seen in Figure 2, there were significant correlations between preoperative logMAR CDVA and both postoperative logMAR CDVA (*r* = 0.63, *P* < 0.001, Figure 2(a)) and changes in logMAR CDVA (*r* = −0.881, *P* < 0.001, Figure 2(b)) for monocular patients.

Table 3 summarizes the statistical linear regression analyses between postoperative questionnaire scores and various explanatory variables for the monocular group. Univariate analysis revealed that preoperative logMAR CDVA, systemic comorbidity, and ocular comorbidity were significant predictors of VFQ-25 scores after cataract surgery (all *P* < 0.05). Age and systemic comorbidities were significantly associated with postoperative SAS scores (both *P* < 0.005). Greater age, the presence of systemic comorbidity, and worse preoperative CDVA were significantly associated with high postoperative SDS scores (all *P* < 0.05). Multivariate analysis revealed that higher postoperative VFQ-25 scores were significantly associated with better preoperative CDVA and the absence of systemic comorbidity (both *P* < 0.05). Age and ocular comorbidity were significantly associated with postoperative SAS scores (both *P* < 0.05). Age and systemic comorbidity remained significant impact factors for SDS scores (both *P* < 0.05).

## 4. Discussion

This study investigated visual outcomes and changes in quality of life and psychological distress of monocular patients after cataract surgery. We also identified how visual outcomes and changes in quality of life and psychological distress related to preoperative visual acuity, systemic comorbidity, ocular comorbidity, and sociodemographic characteristics in monocular patients.

We confirmed that monocular patients experienced the same substantial improvement in CDVA as age- and gender-matched binocular control subjects, despite having worse CDVAs before and after cataract surgery, consistent with findings of previous studies [4, 11, 12, 19, 20]. However, monocular patients in our study experienced greater improvements in CDVA than those reported by Pomberg and Miller [11]. The discrepant findings with respect to CDVA between our study and Miller's study may result from the fact that the CDVA of monocular patients before surgery in our study was worse than that in Miller's study. In China, many people undergo cataract surgery as late as possible until their visual acuity is very poor due to economic reasons or because of insufficient knowledge of cataract. As evidenced by the improvement in CDVA, cataract surgery effectively improves visual acuity in monocular patients.

Many researches have demonstrated that visual impairment due to cataract had negative effects on quality of life, and cataract surgery can improve the quality of life [21–25]. One previous study found that most monocular patients reported incapacity to work and difficulties in performing activities of daily life [26]. Pomberg and Miller reported that monocular patients gained twice the increase in self-reported functional vision as binocular controls, as assessed by the VF-14 [11]. Nevertheless, there have been few studies assessing the impact of cataract in monocular patients in terms of psychosocial aspects. Our data indicate that monocular patients have lower vision-related quality of life before and after cataract surgery than binocular patients do. By contrast, monocular patients obtained about twice the increase in vision-related quality of life as did binocular controls, as assessed by the VFQ-25. This finding is easily explained. Because of the lack of a sighted fellow eye to buffer the handicap caused by cataract, cataract may bring greater direct visual functional impairment to monocular patients, compared to that of binocularly sighted patients. Accordingly, monocular patients with cataract suffer from more severe damage to vision-related quality of life. Because the deficit in vision-related quality of life before surgery is not as great for binocular controls as it is for monocular patients, the improvement they experience is also not as great.

Several studies have mentioned that cataract surgery influenced monocular patients differently in terms of anxiety and depression compared with the effects on binocular patients. Bergwerk and Miller found that the monocular patients were likely to be much more anxious about surgery than 2-eyed patients [4]. Marback et al. reported that patients with monocular vision reported more fear and doubts related to cataract surgical outcomes [27]. In our study, although most of the patients did not meet the criteria for anxiety or depression, more monocular patients expressed anxiety or depression symptoms before cataract surgery than did binocular patients. Furthermore, in terms of SAS and SDS scores, there were larger decreases in monocular patients than in binocular patients. These findings suggest that monocular patients can obtain more improvements in psychological distress, especially anxiety and depression. Consequently, it is necessary for surgeons to pay more attention to the emotional reactions and psychological distress of monocular patients during the perioperative period. We should devote more time than usual to explain the risks and benefits of cataract surgery to help monocular patients cooperate better with surgery and follow-up, for the benefits of recovery and rehabilitation.

There have been many studies which proved that cataract surgery outcomes in monocular patients were generally favorable in the short term [4, 11, 12, 19, 20]. We also found that there were significant associations between the pre- and postoperative logMAR CDVA as well as between the changes in the logMAR CDVA and the postoperative logMAR CDVA in monocular patients. These findings indicate that better preoperative visual acuity may gain better visual outcome at least in the early stages after cataract surgery. From these observations, we presume that it is not in the best interest of monocular patients to delay cataract surgery until the visual handicap from cataract is severe, especially considering that “delaying surgery until the cataract is very advanced may increase surgical risk and slow visual recovery” [28]. Furthermore, the results of our multivariate regression analysis confirmed that better preoperative visual acuity was significantly associated with higher VFQ-25 scores. Thus, early cataract surgery treatment can obtain early and better restoration of quality of life, including fewer workdays lost, fewer accidents, less deterioration in daily living activities, and other financial and psychosocial benefits [29]. Finally, early cataract surgery makes it easier to detect fundus pathology and to treat lesions by laser photocoagulation for patients with fundus comorbidities.

The current study has several limitations. All participants were Han Chinese. This might cause selection bias, and the results may not be generalizable to the entire population of monocular patients with cataract worldwide. In addition, the frequency of recruiting participants was slow because monocular patients are scarce in our cataract department per year. However, the small sample size did not influence the results because all included patients received the same surgery procedures and were assessed in the same way. Besides, follow-up in this study was 1 month. More subjects with long-term postoperative data are required to confirm the impact of cataract surgery on monocular patients in visual and psychosocial aspects.

## 5. Conclusion

Our findings indicate that monocular patients gain more improvements in visual acuity, quality of life, and psychological distress than binocular patients do. It is not a better choice for monocular patients to delay cataract surgery until the cataract becomes severe. It is important for ophthalmologists to take the benefits in quality of life and psychosocial aspects into consideration when making surgery decision, and it is of significance for doctors to pay more attention to the psychological distress of monocular patients with cataract and provide proper counseling and instruction to help the patients cooperate with surgery and follow-up so as to promote their postoperative rehabilitation.

## Figures and Tables

**Table 1:** Sociodemographic and clinical characteristics of study subjects.

Variables	Monocular group	Binocular group	*P* values
Mean age (*y*) ± SD	63.73 ± 11.56	67.08 ± 8.45	0.143
Gender, *n* (%)			
Male	16 (40)	18 (45)	0.651
Female	24 (60)	22 (55)	
Education level			
Junior high school or below	19 (47.5)	17 (42.5)	0.653
Senior high school or higher	21 (52.5)	23 (57.5)	
Residential environment			
Urban	23 (57.5)	36 (90)	0.001^*∗*^
Rural	17 (42.5)	4 (10)	
Systemic comorbidity			
Yes	18 (45)	17 (42.5)	0.822
No	22 (55)	23 (57.5)	
Ocular comorbidity			
Yes	21 (52.5)	10 (25)	0.012^*∗*^
No	19 (47.5)	30 (75)	

^*∗*^ *P* < 0.05.

**Table 2:** Corrected distance visual acuity (CDVA) before surgery and at the 1-month postoperative examination.

	Monocular group (mean ± SD)	Binocular group (mean ± SD)	*P* values
Preoperative logMAR CDVA	0.91 ± 0.60	0.59 ± 0.37	0.01^*∗*^
Postoperative logMAR CDVA	0.22 ± 0.21	0.06 ± 0.08	<0.001^*∗*^
logMAR CDVA change	−0.68 ± 0.48	−0.53 ± 0.38	0.103

^*∗*^ *P* < 0.05.

**Table 3:** Linear regression analysis of factors associated with postoperative NEI-VFQ-25 composite scores, SAS scores, and SDS scores for the monocular group.

Variable	NEI-VFQ-25 composite scores		SAS scores		SDS scores	
	*β* coefficient (95% CI)	*P* values	*β* coefficient (95% CI)	*P* values	*β* coefficient (95% CI)	*P* values
Univariate analysis						
Preoperative logMAR CDVA	−11.01 (−15.76 to −6.27)	<0.001^*∗*^	0.61 (−0.58 to 1.79)	0.307	2.52 (0.19 to 4.86)	0.035^*∗*^
Age (*y*)	−0.33 (−0.74 to −0.09)	0.121	0.12 (0.05 to 0.20)	0.002^*∗*^	0.26 (0.10 to 0.41)	0.002^*∗*^
Gender: male	3.97 (−5.78 to 13.72)	0.414	−0.37 (−2.32 to 1.57)	0.700	0.05 (−3.98 to 4.08)	0.981
Education level: senior high school or higher	−6.21 (−15.47 to 3.05)	0.182	0.81 (−1.06 to 2.67)	0.386	1.50 (−2.26 to 5.44)	0.408
Residential environment: urban	−3.81 (−13.24 to 5.62)	0.417	1.14 (−0.70 to −2.99)	0.216	1.41 (−2.45 to 5.28)	0.463
Systemic comorbidity: yes	−9.78 (−18.74 to −0.83)	0.033^*∗*^	2.52 (0.84 to 4.20)	0.004^*∗*^	6.22 (2.95 to 9.49)	<0.001^*∗*^
Ocular comorbidity: yes	−10.91 (−20.00 to −1.81)	0.0208	1.60 (−0.27 to 3.47)	0.092^*∗*^	3.80 (−0.01 to −7.61)	0.051^*∗*^
Multivariate analysis						
Preoperative logMAR CDVA	−9.58 (−14.45 to −4.71)	<0.001^*∗*^	—	—	1.52 (−0.47 to 3.52)	0.129
Age (*y*)	—	—	0.10 (0.02 to 0.18)	0.014^*∗*^	0.18 (0.03 to 0.32)	0.020^*∗*^
Systemic comorbidity: yes	−8.16 (−15.19 to −1.13)	0.024^*∗*^	1.41 (−0.30 to 3.12)	0.102	4.06 (0.84 to 7.28)	0.015^*∗*^
Ocular comorbidity: yes	−3.92 (−11.80 to 3.96)	0.318	1.58 (−0.002 to 3.15)	0.0497^*∗*^	2.63 (0.61 to 5.87)	0.107

Variables with *P* values <0.1 in univariate analysis were entered into the multivariate analysis. CDVA = corrected distance visual acuity. ^*∗*^*P* < 0.05.

## Data Availability

The data are available from the corresponding author upon request.
